# Bioinformatical Analysis of Organ-Related (Heart, Brain, Liver, and Kidney) and Serum Proteomic Data to Identify Protein Regulation Patterns and Potential Sepsis Biomarkers

**DOI:** 10.1155/2018/3576157

**Published:** 2018-03-21

**Authors:** Andreas Hohn, Ivan Iovino, Fabrizio Cirillo, Hendrik Drinhaus, Kathrin Kleinbrahm, Lennert Boehm, Edoardo De Robertis, Jochen Hinkelbein

**Affiliations:** ^1^Department of Anaesthesiology and Intensive Care Medicine, University Hospital of Cologne, Kerpener Str. 62, 50937 Cologne, Germany; ^2^Department of Neurosciences, Reproductive and Odontostomatological Sciences, University of Naples “Federico II”, Via S. Pansini 5, 80131 Napoli, Italy; ^3^Department of Anaesthesiology and Intensive Care Medicine, “G. Moscati” Hospital, Via Gramsci 1, 81031 Aversa, Italy

## Abstract

During the last years, proteomic studies have revealed several interesting findings in experimental sepsis models and septic patients. However, most studies investigated protein alterations only in single organs or in whole blood. To identify possible sepsis biomarkers and to evaluate the relationship between protein alteration in sepsis affected organs and blood, proteomics data from the heart, brain, liver, kidney, and serum were analysed. Using functional network analyses in combination with hierarchical cluster analysis, we found that protein regulation patterns in organ tissues as well as in serum are highly dynamic. In the tissue proteome, the main functions and pathways affected were the oxidoreductive activity, cell energy generation, or metabolism, whereas in the serum proteome, functions were associated with lipoproteins metabolism and, to a minor extent, with coagulation, inflammatory response, and organ regeneration. Proteins from network analyses of organ tissue did not correlate with statistically significantly regulated serum proteins or with predicted proteins of serum functions. In this study, the combination of proteomic network analyses with cluster analyses is introduced as an approach to deal with high-throughput proteomics data to evaluate the dynamics of protein regulation during sepsis.

## 1. Introduction

Proteomic studies and broad analyses of protein alterations in experimental and clinical sepsis allow evaluating the systemic host response to a hit or injury and offer comprehensive information about the complex host response to infection [1]. Compared with genetic analyses, proteomics can give direct insight into protein expression and not only in an indirect way as would be possible by studying gene regulation. As secreted proteins are signalling systems which convert genetic signals into enzymatic activity, it might be advantageous to study protein alterations [1]. Experience with proteomics in sepsis has already revealed several interesting findings in both experimental models and septic patients [2]. A small clinical study in septic shock patients, for example, demonstrated that proteomic analysis is a feasible tool to exclude early alterations in protein expression and that there are specific protein alterations between survivors and nonsurvivors in an early stage of septic shock [3].

Furthermore, other proteomic studies identified peptides as possibly useful sepsis biomarkers [4, 5]. In a septic mouse animal model, dynamic changes of tissue-specific septic protein profiles in blood plasma could be detected using proteomic analysis [6]. Most studies investigated protein alterations only in single organs or in whole blood [1]. However, sepsis represents a continuum ranging from simple infection and bacteraemia to life threatening septic shock with multiple organ dysfunction. As sepsis is a highly dynamic process, protein alterations may vary during the different stages of the disease [7]. Compared with genetic analyses, proteomics can give direct insight into protein expression and not only in an indirect way as would be possible by studying gene regulation.

To identify possible protein regulation patterns and to evaluate the interaction between protein alteration in sepsis affected organs and blood, proteomics data from the heart, brain, liver, kidney, and serum from previous studies was analysed [8–12].

Modern technologies make it possible to identify and quantify a large amount of different proteins in proteomic experiments. Thus, big data analyses have become a bottleneck and represent a great challenge in proteomics [13]. In this study, protein network analyses in combination with cluster analyses are described as a possible approach to deal with high-throughput proteomic data.

## 2. Material and Methods

### 2.1. Experimental Sepsis Model and Proteomic Data from Previous Studies

In five previous studies, male Wistar rats were randomly assigned to a sepsis group (cecal ligation and puncture, CLP) or a control group (sham) [8–11, 14]. Surviving rats were sacrificed 12, 24, or 48 hours after sepsis induction. Organs and serum were removed after decapitation and prepared for proteomic analysis. Proteins were separated using 2D gel electrophoresis (2D-DIGE). Each spot in the 2D-DIGE was matched to a corresponding spot in a reference gel, which was created as a virtual PC-generated averaged gel. Then, normalized spot volumes between the sham and sepsis groups at each respective time point were compared. In addition to a statistically significant difference between the spots, differences were considered biologically relevant if the protein expression factors (induction factor [IF]) changed more than twofold (IF < 0.5 or IF > 2). This helps ensure that regulated expression has actual biological significance, thus making the changes more likely to affect cellular functions. The IF in relation to the sham group was calculated by dividing the mean normalized spot volumes in both groups. A value of 2.0 therefore indicates a twofold increase, and a value of 0.5 indicates a twofold decrease.

Significantly altered proteins were identified by mass spectrometry (MALDI-TOF MS) and used for further bioinformatical analysis to identify underlying networks, signalling cascades, and pathways affected.

### 2.2. Stepwise Bioinformatical Approach

In summary, as a first step, statistically significantly regulated proteins from blood and organ tissues of previous studies were identified and analysed by network analyses (GeneMania®). Afterwards, those statistically significant proteins were grouped (12, 24, and 48 hrs) using a hierarchical cluster analysis (Perseus®). As a third step, proteins of similarly early upregulated clusters underwent further network analysis to evaluate possible corresponding proteins or functions in blood and organ tissues. This approach to deal with pooled proteomic data is described in detail below.

### 2.3. Network Analysis of Proteins (GeneMania)

Sixty proteins from sepsis related organs (liver, kidney, heart, and brain) and twenty proteins from a serum analysis which were significantly altered, at least at one time point (12, 24, and 48 hours), were used for further bioinformatical analysis to identify underlying networks, signalling cascades, and pathways affected.

Biological functions of statistically significantly regulated proteins were identified using functional network analysis. GeneMania (http://www.genemania.org/) is a tool that helps predict interactions and function of genes in terms of network and, when available, of pathway [15, 16]. It gives the possibility of customizing the network and allows choosing data sources or highlighting specific functions, with a more comfortable graphic experience [15]. It is developed and continually updated by the University of Toronto and is funded by the Ontario Ministry of Research and Innovation. GeneMania knowledge is based on data from large databases, which comprehend Gene Expression Omnibus, BioGRID, EMBL-EBI, Pfam, Ensembl, Mouse Genome Informatics, the National Center for Biotechnology Information, InParanoid, and Pathway Commons [15, 16]. It was developed for making predictions about gene or protein function based on a query of list of proteins that share a function of interest. The software allows taking advantage of the persistent improvement and proliferation of high-throughput genomics and proteomics datasets by making up-to-date predictions of their interaction with other genes or proteins [15, 16].

As these software programs use different algorithms, we decided to perform the bioinformatical analyses with all of them in order to retrieve the highest number of predicted interactions, maintaining an acceptable level of confidence (0.400).

The associated functions detected by the software were downloaded in TAB-separated-values format and exported to Microsoft Excel® (Microsoft, Redmond, USA; version 2007) where they were filtered in subgroups which were reanalysed using GeneMania.

### 2.4. Hierarchical Cluster Analysis

Heat maps are an efficient method of visualizing complex datasets organized as matrices [17]. Perseus (Max Planck Institute of Biochemistry, Martinsried, Germany; v. 1.5.8.5) is a holistic software platform that allows continuous expansion of scalable analytical tools, their smooth integration, and reusability while providing the user with explicit documentation of the analysis steps and parameters [18]. Quantitative information concerning proteins that had statistically significant altered expression at 12, 24, and 48 hours from the induction of sepsis was converted to TSV (Tab-Separated Values) text file using Microsoft Excel (Microsoft, Redmond, USA; version 2007). Each value was reported as fold change in comparison to the sham group values, so that a positive number represents a higher expression of a spot at 12, 24, or 48 hours while a negative number represents lower expression of a spot at 12, 24, or 48 hours. In this format the data were analysed using the free software Perseus (Max Planck Institute of Biochemistry, Martinsried, Germany; v. 1.5.8.5) which performed the *Z*-scoring and, consequently, the hierarchical cluster analysis. The resulting heat map can be interpreted on the basis of colour intensity. In our case, a red brick represents a protein whose expression at a particular time was increased when compared to the value of the same protein in the sham group at that time.

### 2.5. Identification of Regulation Pathways and Biomarker Candidates

On the basis of the cluster analysis, further subgroup network analyses of similarly upregulated proteins at 12 hours or 12 and 24 hours after sepsis induction in sepsis related organs (liver, kidney, heart, and brain) were performed to find regulation patterns and identify possible biomarkers.

## 3. Results 

### 3.1. Biologically Statistically Significantly Regulated Proteins

Collecting data from the 5 previous studies [8–12], 80 statistically significant altered proteins (a total of 113 total spots) from sepsis related organs and serum were identified.

**Figure 1:**
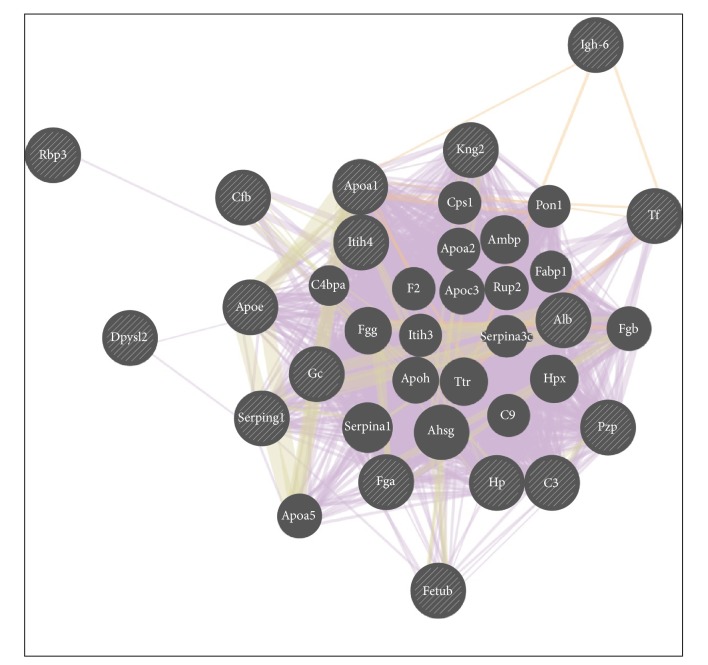
Network analysis of serum proteins. In a GeneMania network analysis, each circle represents a gene. The input proteins/genes are depicted as striped circles of the same size, while the monochromatic circles, whose size is proportional to the number of interactions according to the software, can be considered “relevant” related genes found by GeneMania searching in many large, publicly available biological datasets (including protein-protein, protein-DNA, and genetic interactions, pathways, reactions, gene and protein expression data, protein domains, and phenotypic screening profiles). Lines linking different circles can be distinguished from their colour; mainly violet represents coexpression (when expression levels are similar across conditions in a gene expression study); light orange represents predicted functional relationships between genes.

Using GeneMania, separate network analyses regarding serum proteins (Figure 1) and regarding sepsis related organs (liver, kidney, heart, and brain) (Suppl.  Figure 1) were performed. The detected organ-related functions were subsequently filtered. From the original 159 functions, we found 38 functions filtered for prevalence (arbitrary cutoff at 12%) (Table 1) and 51 functions filtered by absolute number (cutoff ≥ 7) (Suppl.  Table 1).

Most of the functions were associated with oxidoreductive activity and cell energy generation or metabolism (ATP production, tricarboxylic metabolism, glycolysis, gluconeogenesis, cell respiration, etc.) and nucleotide or nucleoside metabolism. One-third of the proteins found are usually located in the mitochondria.

The functions identified with statistically significant altered serum proteins using 2% as cutoff for prevalence are shown in Table 2. Functions identified with 6 for absolute numbers as cutoffs are shown in Suppl.  Table 2. Most of the functions for the serum proteins were associated with lipoproteins metabolism and, to a minor extent, with coagulation, inflammatory response, and organ regeneration.

### 3.2. Hierarchical Cluster Analyses and Heat Maps

**Figure 2:**
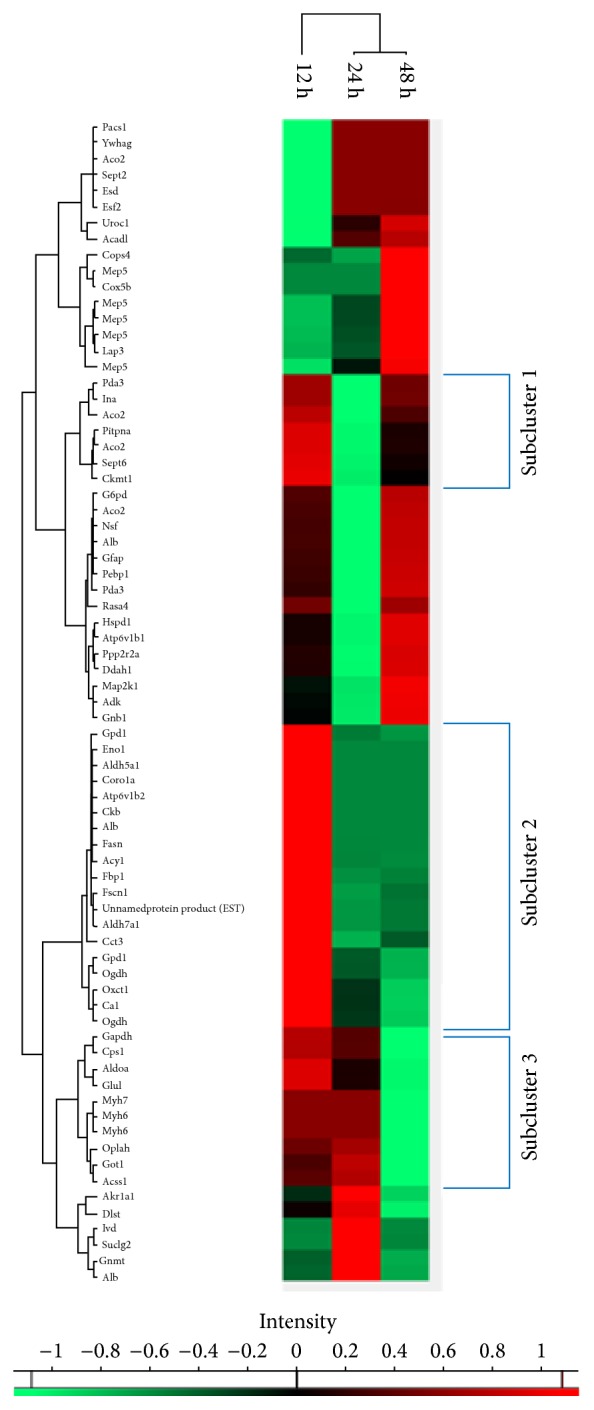
Heat map of the hierarchical cluster analysis of significantly regulated proteins of sepsis related organs. Three subclusters with significantly upregulated proteins at 12 or 12 and 24 hours are highlighted. A brick can progressively become darker up to a completely black one that would represent a fold change equal to 1 (therefore, no change between sepsis and sham groups). On the contrary, a green brick represents a protein whose expression at a particular time was decreased when compared to the value of the same protein in the sham group at that time.

Quantitative information concerning proteins that had statistically significant altered expression in the liver, kidney, heart, and brain at 12, 24, and 48 hours from the induction of sepsis was analysed using Perseus (Max Planck Institute of Biochemistry, Martinsried, Germany; v. 1.5.8.5) which performed the hierarchical cluster analysis (Figure 2).

**Figure 3:**
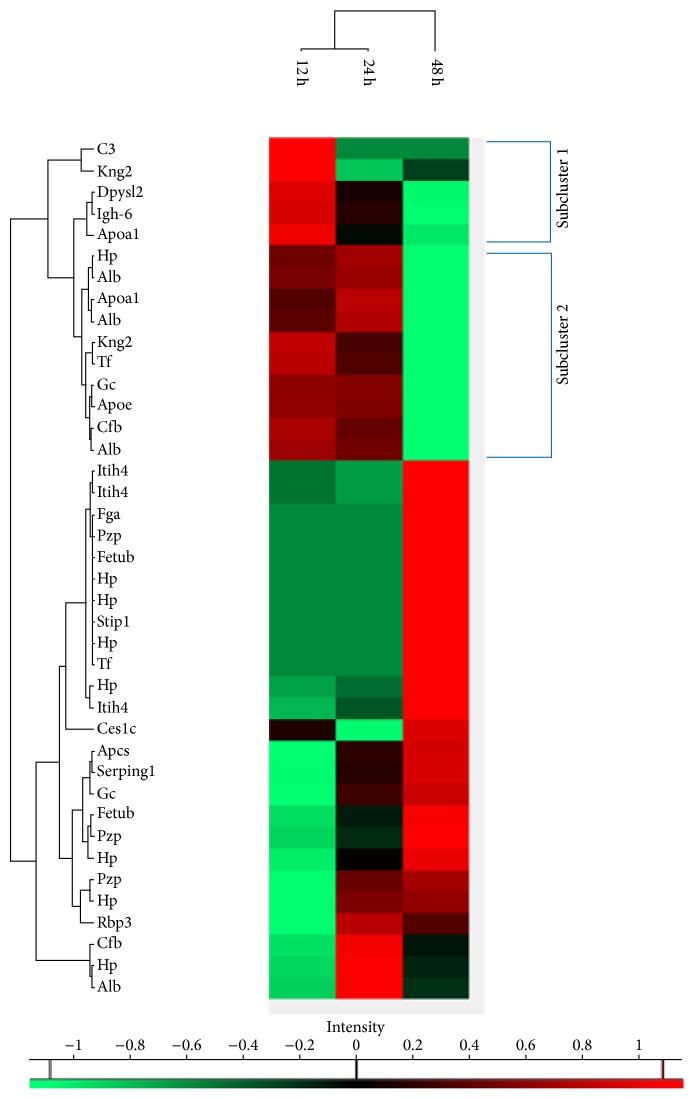
Heat map of the hierarchical cluster analysis of significantly regulated serum proteins. Two subclusters with significantly upregulated proteins at 12 or 12 and 24 hours are highlighted. A brick can progressively become darker up to a completely black one that would represent a fold change equal to 1 (therefore, no change between sepsis and sham groups). On the contrary, a green brick represents a protein whose expression at a particular time was decreased when compared to the value of the same protein in the sham group at that time.

The cluster analysis revealed several groups of regulation patterns with different combinations of proteins up/downregulated or unchanged at different time points. Three subclusters of similarly upregulated proteins at 12 or 12 and 24 hours were identified. Since these early upregulated subclusters may contain possible candidates for sepsis biomarkers, further network analyses were conducted for these subgroups highlighted in Figure 3.

In the same way, a cluster analysis of statistically significantly regulated serum proteins was performed (Figure 3). In this analysis, two subgroups of upregulated proteins at 12 hours or 12 and 24 hours could be identified. Comparing likewise regulated proteins from sepsis related organs and serum, no concordance of proteins could be detected.

### 3.3. Network Analyses of Similarly Regulated Proteins

The subclusters of similarly up- and downregulated proteins in the first 24 hours after sepsis induction for both sepsis related organs and serum underwent further GeneMania analyses to identify networks and predicted proteins within these networks and their associated functions. By identifying predicted proteins, we expected a higher likelihood of finding statistically significantly regulated proteins both in organ tissues and in serum. For subcluster 1 in the organ tissue cluster analysis, we found no network using GeneMania.

The network for subcluster 2 revealed 19 functions filtered by absolute number (cutoff ≥ 5) and 17 functions filtered by prevalence (cutoff ≥ 10%) (Suppl. Tables 3 and 4). Most of the functions in this subcluster were related to oxidoreductive activity and cell energy generation and metabolism, like in the unselected group of the whole tissues. None of the predicted proteins within the functions and pathways was previously found in serum.

Using the same cutoff values in subcluster 3, 27 functions filtered by absolute number and 20 functions filtered by prevalence were found (Suppl. Tables 5 and 6). Most of the functions regarding this subcluster were related to energy generation and metabolism and to muscle contractile function (heart), and nucleoside metabolism. Similar to subcluster 2, none of the predicted proteins within the functions and pathways was previously found in the serum.

In serum proteins, a network analysis of subcluster 1 (Figure 3) with an upregulated group at 12 hours (C3, Apoa1, Kng2, Dpysl2, and Igh-6) was not possible because the number of proteins involved was too low. Therefore, subclusters 1 (see above) and 2 (Hp, Alb, Apoa1, Kng2, Tf, Gc, Apoe, and Cfb) were analysed together and most of the functions were related to lipid metabolism or lipid transport and to a lower extent associated with immune response (Suppl. Tables 7 and 8).

## 4. Discussion

In this study, proteomic data of various experiments all using the same experimental sepsis model (i.e., cecal ligation and puncture, CLP) were analysed using bioinformatical methods to identify protein regulation patterns altered by sepsis [8–12]. To the best of our knowledge, this is the first study which compares proteomic data from a broad set of organs during sepsis to associated protein regulation patterns and pathways in serum. Furthermore, we used protein network analysis in combination with hierarchical cluster analysis to deal with large proteomic data. The combination of cluster analysis and network analysis is well established in proteomic studies. However, so far, this approach was not described in an animal model to analyse septic induced protein alterations at different time points.

### 4.1. Functions of Significantly Regulated Proteins

The study reveals several major findings. By using protein network analysis software (GeneMania), we demonstrated that most of the statistically significantly regulated proteins from the heart, liver, kidney, and brain were associated with oxidoreductive activity, cell energy generation or metabolism (ATP production, tricarboxylic metabolism, glycolysis, gluconeogenesis, cell respiration, etc.), and nucleotide or nucleoside metabolism (Table 1 and Suppl.  Table 2). Most of the functions of statistically significantly regulated serum proteins were related to lipoproteins metabolism and, to a minor extent, to coagulation, inflammatory response, and organ regeneration.

It appears plausible that in the clinical setting of sepsis there is an alteration of proteins involved in energy generation in tissues since an imbalance between oxygen delivery and consumption is a hallmark of sepsis and particularly septic shock [19]. Therefore, it is conceivable that expression of proteins related to energy generation might be a compensatory mechanism to account for intracellular hypoxia. Previous studies also showed an association between sepsis and organ failure. Future studies could investigate whether this is a feature specific to sepsis and septic shock or common to different causes of shock and hypoxia.

Concerning lipoprotein expression, which was found to be altered in serum in our study, there is an evolving interest in the use of lipoproteins, especially high-density lipoprotein, both as a biomarker [20, 21] and as a potential therapeutic approach in sepsis [21, 22].

### 4.2. Course of Protein Alterations

Hierarchical cluster analysis confirmed that protein regulation in sepsis related organs and tissues underlies a dynamic process. We found that proteins can be up- or downregulated or even remain unchanged at different time points (12 hours, 24 hours, or 48 hours) after induction of sepsis. Regarding the early phase of sepsis, that is, up to 24 hours after sepsis induction, three subclusters of organ proteins were identified which were upregulated at 12 or at 12 and 24 hours (Figure 3). Subclusters were defined based on the hypothesis that statistically significantly upregulated proteins in organ tissues can probably be found simultaneously in blood. We focused on the early phase of sepsis up to 24 hours after sepsis induction as from a clinical point of view, a timely diagnosis of sepsis is crucial. Proteins of these subclusters in principle could be candidates for early sepsis biomarkers if detected in blood.

### 4.3. Congruency in Regulation between Tissue and Serum

Another major finding of our analysis was that proteins in early upregulated subclusters of the serum (Figure 3) did not correspond to tissue proteins of different organs analysed. Also, functions in upregulated subclusters in serum identified by GeneMania network analysis did not correspond to functions in early upregulated organ tissue clusters. In serum, functions were related to lipoprotein metabolism and, to a minor extent, to coagulation, inflammatory response, and organ regeneration, whereas in organ tissues most functions were associated with energy generation and metabolism and with muscle contractile function (heart) and nucleoside metabolism (Suppl. Tables 5 and 6). Finally, predicted proteins from network analyses of organ tissue did not correlate with significantly regulated serum proteins or with predicted proteins of serum functions.

### 4.4. Evaluation of the Bioinformatical Approach

In our bioinformatical analysis we sought to assess if the dynamic process of sepsis associated alterations in tissue proteome is reflected in serum proteome changes. Several subclusters of early upregulated tissue proteins could be detected, which are possible interesting candidates as sepsis biomarker, if detected in blood. Furthermore, functions and pathways in organ tissues associated with early upregulated protein clusters could be compared to altered functions in blood. However, none of the tissue proteins was found in the serum and, moreover, even none of the predicted proteins from the GeneMania network functions correlated with serum proteins. Even though no identical proteins were detected in the serum as well as in the organ tissues, our bioinformatical approach could be helpful for our understanding of the pathophysiology of sepsis. For example, the cluster analyses revealed which proteins and functions were regulated at different stages during the course of sepsis. Furthermore, one-third of statistically significantly regulated proteins can be found in the mitochondria, underlining the importance of alteration of mitochondrial functions and even mitochondrial damage in the host response to sepsis [12, 23–25].

Even though no common protein was found in the serum as well as in organ tissue, this does not necessarily mean that the detected proteins might not be potential candidates of sepsis biomarkers. Probably, the organ-related proteins were not found in the serum because they were under the detection limit and more sensitive techniques are needed. By using network analyses we were able to predict proteins possibly involved in functions and pathways of upregulated clusters. As a result of this, the number of possible candidates for biomarkers could be increased. The detection of a single protein or a set of proteins, upregulated in organ tissue as well as in serum, would implicate further research in those proteins.

In blood plasma, numerous tissue proteins can be found. However, most of them do not contribute to the genuine blood plasma functions [26]. Currently, there is limited knowledge on the regulation of the blood plasma proteome and it is unknown to what extent various tissues can affect blood plasma protein composition in sepsis [6].

In a recent septic mouse model, the authors introduced an MS-based strategy to monitor the dynamics of tissue and cell-specific proteins in the blood plasma and constructed a proteome-wide tissue atlas to demonstrate how the surrounding tissue and cells influence the blood plasma in severe infectious diseases [6]. In their study, only one single time point at 48 hours after sepsis induction was analysed, whereas in our study we sought to identify early time-dependent correlations between changes in organ and blood proteome using a hierarchical cluster analysis. Hierarchical cluster analysis turned out to be useful in both detection of possible biomarkers and protein regulation patterns in clinical or experimental sepsis research [27–30].

In a recent review article the authors stated that “in case of the proteomic investigation, the challenges occur at all levels ranging from sample preparation and data gathering over the raw data integration and database searching to the functional interpretation of large datasets” [31]. Thus, our bioinformatical analysis might be a promising method of how to deal with large proteomic data and complex interactions and functions. In future, proteomic techniques will steadily improve and data quantities will increase. Thus, new methods are required, helping us to interpret these results. In this context, our study should be rather interpreted as hypothesis generating rather than definitive. Nonetheless, there are no current standards on settings or cutoff levels for network analysis software. For the present study, we used default settings of the network analysis software. Cutoffs for proteins and functions were defined arbitrarily only to find a pragmatical balance between finding relevant sepsis related functions and eliminating nonspecific proteins. Of course, these settings and cutoffs might have influenced the results in our analysis and further analyses should aim for this. Likewise, protein clusters in our study were defined from a clinical point of view. Future studies should evaluate the most appropriate selection algorithms and software settings and should compare different network analysis software programs. Nonetheless, every study is unique and software settings and cutoffs also depend on the type of analysis and the hypotheses.

### 4.5. Limitations

Some limitations of our study have to be mentioned. Statistically significantly regulated tissue proteins from different organs were mixed in the network analyses. Thus, we cannot be sure that the derived functions and pathways in fact correspond to these functions in the respective organs. However, the previous organ proteomics analyses of this sepsis model confirm that most of the functions are associated with energy metabolism, mitochondrial function, and lipid metabolism [8–12].

The number of functions presented in this analysis was limited by using arbitrary cutoffs for prevalence and the absolute number of proteins involved in the network. By this, functions were identified in which only a representative number of proteins was present.

Interestingly, we found no typical acute phase proteins in our analysis. This probably depends on the technical limitations of proteomic analyses. As common inflammation biomarkers are relatively small proteins and concentration even after upregulation might be low, this could explain why those typical proteins were missed in our analysis. With further advances in proteomic techniques and more sensitive methods, small and low concentrated proteins might also be detected in future.

## 5. Conclusion

In summary, in our stepwise comparison of dynamic organ tissue proteome changes to serum proteome changes we were able to demonstrate that regulation patterns in organ tissues as well as in serum are highly dynamic. Subclusters of proteins can be upregulated or downregulated or even remain undifferentiated at different stages of sepsis. The main functions and pathways affected in the tissue proteome were oxidoreductive activity, cell energy generation, or metabolism, whereas in the serum proteome, functions were associated with lipoproteins metabolism and, to a minor extent, with coagulation, inflammatory response, and organ regeneration. Using hierarchical cluster analyses and functional network analyses (GeneMania) including predicted network proteins, we were not able to detect correlating proteins or functions in organ tissues and blood. Furthermore, we were not able to identify promising candidates for sepsis biomarkers. Nonetheless, this analysis provides new insights into protein regulation during sepsis and this bioinformatical approach could be helpful to deal with high-throughput proteomic data.

## Figures and Tables

**Table 1:** Thirty-eight functions filtered by prevalence (cutoff ≥ 12%) from the original 159 functions derived from GeneMania network analysis of the whole dataset without the serum proteins. Column 1 shows the functions names. Columns 2 and 3 show, respectively, the number of annotated genes in the displayed network and the number of genes with that annotation in the genome. In column 5, names in bold letters represent the genes predicted by the software.

Function	Genes in network	Genes in genome	Ratio	Names
NADH metabolic process	6	12	50.00%	gpd1, dlst, ogdh, **mdh2, mdh1, aldob**
Oxaloacetate metabolic process	4	11	36.36%	got1, **mdh1, mdh2, pc**
Tricarboxylic acid cycle	5	15	33.33%	dlst, ogdh, aco2, suclg2, **mdh2**
Tricarboxylic acid cycle enzyme complex	3	11	27.27%	dlst, ogdh, suclg2
NAD metabolic process	6	24	25.00%	gpd1, dlst, ogdh, **aldob, mdh2, mdh1**
Aerobic respiration	5	25	20.00%	dlst, ogdh, aco2, suclg2,** mdh2**
Fatty-acyl-CoA binding	3	15	20.00%	acadl, pitpna, **acadm**
Succinate metabolic process	2	10	20.00%	aldh5a1, suclg2
Pentose-phosphate shunt	2	10	20.00%	g6pd, **taldo1**
Ribonucleoside diphosphate biosynthetic process	2	10	20.00%	**atp5a1, atp5b**
Pentose metabolic process	2	10	20.00%	g6pd, **taldo1**
NADPH regeneration	2	10	20.00%	g6pd, **taldo1**
2-Oxoglutarate metabolic process	3	16	18.75%	got1, dlst, ogdh
Nicotinamide nucleotide metabolic process	8	43	18.60%	gpd1, dlst, ogdh, g6pd, **mdh2, mdh1, taldo1, aldob**
Pyridine nucleotide metabolic process	8	43	18.60%	gpd1, dlst, ogdh, g6pd, **mdh2, mdh1, taldo1, aldob**
MHC class I protein binding	2	11	18.18%	**atp5a1, atp5b**
ADP metabolic process	2	11	18.18%	**atp5a1, atp5b**
Positive regulation of glycolysis	2	11	18.18%	gpd1, **mif**
Oxidoreductase activity, acting on the aldehyde or oxo group of donors, NAD or NADP as acceptor	4	25	16.00%	aldh5a1, ogdh, gapdh, aldh7a1
Monosaccharide catabolic process	10	63	15.87%	aldoa, akr1a1, gapdh, eno1, g6pd, fbp1, gpd1, **taldo1, mif, aldob**
Glucose catabolic process	9	57	15.79%	fbp1, gpd1, aldoa, gapdh, eno1, g6pd, **taldo1, mif, aldob**
Neurotransmitter metabolic process	3	19	15.79%	aldh5a1, glul, pebp1
Pyridine-containing compound metabolic process	8	51	15.69%	gpd1, dlst, ogdh, g6pd, **mdh2, mdh1, taldo1, aldob**
Monosaccharide biosynthetic process	8	51	15.69%	gnmt, akr1a1, gapdh, g6pd, fbp1, gpd1, **pc, acadm**
Oxidoreduction coenzyme metabolic process	8	51	15.69%	gpd1, dlst, ogdh, g6pd, **mdh2, mdh1, taldo1, aldob**
Acetyl-CoA metabolic process	4	26	15.38%	acss1, fasn, **dlat, acaa2**
Glycolysis	7	46	15.22%	fbp1, gpd1, aldoa, gapdh, eno1,** mif, aldob**
Glutamate metabolic process	3	20	15.00%	aldh5a1, got1, glul
Hexose catabolic process	9	61	14.75%	fbp1, gpd1, aldoa, gapdh, eno1, g6pd, **taldo1, mif, aldob**
Gluconeogenesis	6	44	13.64%	gnmt, gapdh, fbp1, gpd1, **pc, acadm**
Dicarboxylic acid metabolic process	10	76	13.16%	gnmt, ogdh, glul, suclg2, aldh5a1, got1, dlst, **mdh1, mdh2, pc**
Hexose biosynthetic process	6	46	13.04%	gnmt, gapdh, fbp1, gpd1, **pc, acadm**
Purine nucleoside triphosphate biosynthetic process	3	23	13.04%	adk, aldoa, **atp5b**
Oxidoreductase activity, acting on the aldehyde or oxo group of donors	4	31	12.90%	aldh5a1, ogdh, gapdh, aldh7a1
Single-organism carbohydrate catabolic process	11	90	12.22%	cps1, aldoa, akr1a1, gapdh, eno1, c6pd, fbp1, gpd1, **taldo1, mif, aldob**
Regulation of glycolysis	3	25	12.00%	fbp1, gpd1, **mif**
Proton-transporting two-sector ATPase complex	3	25	12.00%	atp6v1b1, **atp5a1, atp5b**
Hydro-lyase activity	3	25	12.00%	uroc1, aco2, eno1

**Table 2:** Network analysis serum functions prevalence. Twenty-nine functions filtered by prevalence (cutoff ≥ 2%) from the original 166 functions derived from GeneMania® network analysis of the serum-protein dataset. Column 1 shows the functions names. Columns 2 and 3 show, respectively, the number of annotated genes in the displayed network and the number of genes with that annotation in the genome. In column 5, names in bold letters represent the genes predicted by the software.

Function	Genes in network	Genes in genome	Ratio	Names
Blood microparticle	22	97	22.68%	apcs, hp, c3, tf, apoa1, cfb, apoe, serping1, fga, alb, itih4, gc, **ahsg, c9, hpx, fgb, pon1, ambp, apoa2, f2, c4bpa, fgg**
Glycerolipid metabolic process	9	211	4.27%	c3, apoa1, apoe, **apoa5, apoh, pon1, cps1, apoa2, apoc3**
Phospholipid binding	9	222	4.05%	apoe, apoa1, **apoa5, apoh, fabp1, pon1, cps1, apoa2, apoc3**
Negative regulation of hydrolase activity	9	264	3.41%	fetub, kng2, apoa1, serping1, **serpina1, fabp1, ambp, apoa2, apoc3**
Lipid transport	8	174	4.60%	apoe, apoa1, **apoa5, apoh, fabp1, pon1, apoa2, apoc3**
Regeneration	8	184	4.35%	fga, hp, apoa1, apoe, **apoa5, ahsg, apoa2, apoh**
Enzyme inhibitor activity	8	197	4.06%	fetub, apoa1, serping1, **serpina1, ahsg, ambp, apoa2, apoc3**
Wound healing	8	287	2.79%	fga, c3, apoe, **apoa5, c9, fgb, f2, apoh**
High-density lipoprotein particle	7	15	46.67%	apoe, apoa1, **apoa5, apoh, pon1, apoa2, apoc3**
Plasma lipoprotein particle	7	19	36.84%	apoe, apoa1, **apoa5, apoh, pon1, apoa2, apoc3**
Protein-lipid complex	7	20	35.00%	apoe, apoa1, **apoa5, apoh, pon1, apoa2, apoc3**
Acylglycerol metabolic process	7	75	9.33%	c3, apoe, **apoa5, apoh, apoc3, apoa2, cps1**
Neutral lipid metabolic process	7	77	9.09%	c3, apoe, **apoa5, apoh, apoc3, apoa2, cps1**
Acute inflammatory response	7	96	7.29%	hp, c3, tf, itih4, serping1, **ahsg, apoa2**
Lipid localization	7	136	5.15%	apoe, apoa1, **apoa5, apoh, fabp1, apoa2, apoc3**
Regulation of lipid metabolic process	7	229	3.06%	c3, apoa1, apoe, **apoa5, fabp1, apoa2, apoc3**
Regulation of body fluid levels	7	246	2.85%	c3, apoe, gc, fga, **c9, f2, apoh**
Extracellular matrix	7	262	2.67%	apcs, alb, tf, rbp3, **f2, apoh, ahsg**
Triglyceride-rich lipoprotein particle	6	14	42.86%	apoe, apoa1, **apoa5, apoh, apoc3, apoa2**
Very-low-density lipoprotein particle	6	14	42.86%	apoe, apoa1, **apoa5, apoh, apoc3, apoa2**
Triglyceride metabolic process	6	67	8.96%	c3, apoe, **apoa5, apoh, apoc3, cps1**
Organ regeneration	6	92	6.52%	hp, apoa1, **apoa5, ahsg, apoa2, apoh**
Blood coagulation	6	110	5.45%	c3, apoe, fga, **c9, f2, apoh**
Hemostasis	6	112	5.36%	c3, apoe, fga, **c9, f2, apoh**
Coagulation	6	115	5.22%	c3, apoe, fga, **c9, f2, apoh**
Negative regulation of endopeptidase activity	6	156	3.85%	fetub, kng2, serping1, **serpina1, fabp1, ambp**
Lipid catabolic process	6	157	3.82%	apoe, **apoa5, fabp1, cps1, apoa2, apoc3**
Negative regulation of peptidase activity	6	159	3.77%	fetub, kng2, serping1, **serpina1, fabp1, ambp**
Steroid metabolic process	6	200	3.00%	gc, apoa1, apoe, **apoc3, pon1, apoa2**
Alcohol metabolic process	6	211	2.84%	gc, apoa1, apoe, **apoc3, pon1, apoa2**
Regulation of endopeptidase activity	6	276	2.17%	fetub, kng2, serping1, **serpina1, fabp1, ambp**
Organic anion transport	6	279	2.15%	dpysl2, apoa1, apoe, **apoc3, fabp1, apoa2**
Regulation of peptidase activity	6	288	2.08%	fetub, kng2, serping1, **serpina1, fabp1, ambp**

## Supplementary Materials

Supplementary Materials Suppl. Table 1: fifty-one functions filtered by absolute number (cutoff ≥ 7) from the original 159 functions derived from GeneMania network analysis of the whole dataset without the serum proteins. Column 1 shows the functions names. Columns 2 and 3 show, respectively, the number of annotated genes in the displayed network and the number of genes with that annotation in the genome. In column 5, names in bold letters represent the genes predicted by the software. Suppl. Table 2: network analysis serum functions absolute number. Thirty-three functions filtered by absolute number (cutoff ≥ 6) from the original 166 derived from GeneMania network analysis of the serum-protein dataset. Column 1 shows the functions names. Columns 2 and 3 show, respectively, the number of annotated genes in the displayed network and the number of genes with that annotation in the genome. In column 5, names in bold letters represent the genes predicted by the software. Suppl. Table 3: subcluster 2 (gpd1, eno1, aldh5a1, coro1a, atp6v1b2, ckb, alb, fasn, acy1, fbp1, fscn1, aldh7a1, cct3, gpd1, ogdh, oxct1, and ca1). Seventeen functions filtered by prevalence (cutoff ≥ 10%) from the original 51 functions derived from GeneMania network analysis of the whole dataset without the serum proteins. Column 1 shows the functions names. Columns 2 and 3 show, respectively, the number of annotated genes in the displayed network and the number of genes with that annotation in the genome. In column 5, names in bold letters represent the genes predicted by the software. Suppl. Table 4: subcluster 2 (gpd1, eno1, aldh5a1, coro1a, atp6v1b2, ckb, alb, fasn, acy1, fbp1, fscn1, aldh7a1, cct3, gpd1, ogdh, oxct1, and ca1). Nineteen functions filtered by absolute number (cutoff ≥ 5) from the original 51 functions derived from GeneMania network analysis of the whole dataset without the serum proteins. Column 1 shows the functions names. Columns 2 and 3 show, respectively, the number of annotated genes in the displayed network and the number of genes with that annotation in the genome. In column 5, names in bold letters represent the genes predicted by the software. Suppl. Table 5: subcluster 3 (gapdh, cps1, aldoa, glul, myh6, myh7, oplah, got1, and acss1). Twenty functions filtered by prevalence (cutoff ≥ 10%) from the original 90 functions derived from GeneMania network analysis of the whole dataset without the serum proteins. Column 1 shows the functions names. Columns 2 and 3 show, respectively, the number of annotated genes in the displayed network and the number of genes with that annotation in the genome. In column 5, names in bold letters represent the genes predicted by the software. Suppl. Table 6: subcluster 3 (gapdh, cps1, aldoa, glul, myh6, myh7, oplah, got1, and acss1). Twenty-seven functions filtered by absolute number (cutoff ≥ 5) from the original 90 functions derived from GeneMania network analysis of the whole dataset without the serum proteins. Column 1 shows the functions names. Columns 2 and 3 show, respectively, the number of annotated genes in the displayed network and the number of genes with that annotation in the genome. In column 5, names in bold letters represent the genes predicted by the software. Suppl. Table 7: subcluster of similarly upregulated proteins from the serum-protein dataset (c3, kng2, dpysl2, igh-6, apoa1, hp, alb, tf, gc, apoe, and cfb). Forty-four functions filtered by prevalence (cutoff ≥ 15%) from the original 190 functions derived from GeneMania network analysis of this subcluster dataset. Column 1 shows the functions names. Columns 2 and 3 show, respectively, the number of annotated genes in the displayed network and the number of genes with that annotation in the genome. In column 5, names in bold letters represent the genes predicted by the software. Suppl. Table 8: subcluster of similarly upregulated proteins from the serum-protein dataset (c3, kng2, dpysl2, igh-6, apoa1, hp, alb, tf, gc, apoe, and cfb). Fifty-nine functions filtered by absolute number (cutoff ≥ 5) from the original 190 functions derived from GeneMania network analysis of this subcluster dataset. Column 1 shows the functions names. Columns 2 and 3 show, respectively, the number of annotated genes in the displayed network and the number of genes with that annotation in the genome. In column 5, names in bold letters represent the genes predicted by the software. Suppl. Figure 1: network analysis organs without serum. In a GeneMania network analysis, each circle represents a gene. The input proteins/genes are depicted as striped circles of the same size, while the monochromatic circles, whose size is proportional to the number of interactions according to the software, can be considered as “relevant” related genes found by GeneMania searching in many large, publicly available biological datasets (including protein-protein, protein-DNA, and genetic interactions, pathways, reactions, gene and protein expression data, protein domains, and phenotypic screening profiles). Lines linking different circles can be distinguished from their colour; mainly violet represents coexpression (when expression levels are similar across conditions in a gene expression study); light orange represents predicted functional relationships between genes; light blue represents colocalization (when genes are expressed in the same tissue or proteins found in the same location); light yellow represents shared protein domains (when two gene products have the same protein domain).

## References

[1] Sharma N. K., Salomao R. (2017). Sepsis through the eyes of proteomics: The progress in the last decade. *Shock*.

[2] LaRosa S. P., Opal S. M. (2011). Biomarkers: The Future. *Critical Care Clinics*.

[3] Kalenka A., Feldmann R. E., Otero K., Maurer M. H., Waschke K. F., Fiedler F. (2006). Changes in the serum proteome of patients with sepsis and septic shock. *Anesthesia & Analgesia*.

[4] Paugam-Burtz C., Albuquerque M., Baron G. (2010). Plasma proteome to look for diagnostic biomarkers of early bacterial sepsis after liver transplantation: A preliminary study. *Anesthesiology*.

[5] Hattori N., Oda S., Sadahiro T. (2009). YKL-40 identified by proteomic analysis as a biomarker of sepsis. *Shock*.

[6] Malmström E., Kilsgård O., Hauri S. (2016). Large-scale inference of protein tissue origin in gram-positive sepsis plasma using quantitative targeted proteomics. *Nature Communications*.

[7] Raju M. S., V J., Kamaraju R. S. (2016). Continuous evaluation of changes in the serum proteome from early to late stages of sepsis caused by Klebsiella pneumoniae. *Molecular Medicine Reports*.

[8] Hinkelbein J., Feldmann R. E., Peterka A. (2007). Alterations in cerebral metabolomics and proteomic expression during sepsis. *Current Neurovascular Research*.

[9] Hinkelbein J., Feldmann R. E., Schubert C. (2009). Alterations in rat serum proteome and metabolome as putative disease markers in sepsis. *The Journal of Trauma and Acute Care Surgery*.

[10] Hinkelbein J., Kalenka A., Feldmann R. E. (2009). Early alterations in rat brain protein expression during sepsis. *Der Anaesthesist*.

[11] Hinkelbein J., Kalenka A., Schubert C., Peterka A., Feldmann R. E. (2010). Proteome and metabolome alterations in heart and liver indicate compromised energy production during sepsis. *Protein and Peptide Letters*.

[12] Hinkelbein J., Böhm L., Braunecker S., Adler C., De Robertis E., Cirillo F. (2017). Decreased Tissue COX5B Expression and Mitochondrial Dysfunction during Sepsis-Induced Kidney Injury in Rats. *Oxidative Medicine and Cellular Longevity*.

[13] Rigbolt K. T. G., Vanselow J. T., Blagoev B. (2011). GProX, a user-friendly platform for bioinformatics analysis and visualization of quantitative proteomics data.. *Molecular & cellular proteomics : MCP*.

[14] Hinkelbein J., Feldmann R. E., Kalenka A. (2010). Time-dependent alterations of cerebral proteins following short-term normobaric hyperoxia. *Molecular and Cellular Biochemistry*.

[15] Warde-Farley D., Donaldson S. L., Comes O. (2010). The GeneMANIA prediction server: biological network integration for gene prioritization and predicting gene function. *Nucleic Acids Research*.

[16] Mostafavi S., Ray D., Warde-Farley D., Grouios C., Morris Q. (2008). GeneMANIA: a real-time multiple association network integration algorithm for predicting gene function. *Genome Biology*.

[17] Key M. (2012). A tutorial in displaying mass spectrometry-based proteomic data using heat maps.. *BMC Bioinformatics*.

[18] Tyanova S., Temu T., Sinitcyn P. (2016). The Perseus computational platform for comprehensive analysis of (prote)omics data. *Nature Methods*.

[19] Gotts J. E., Matthay M. A. (2016). Sepsis: pathophysiology and clinical management. *BMJ*.

[20] Cirstea M., Walley K. R., Russell J. A., Brunham L. R., Genga K. R., Boyd J. H. (2017). Decreased high-density lipoprotein cholesterol level is an early prognostic marker for organ dysfunction and death in patients with suspected sepsis. *Journal of Critical Care*.

[21] Morin E. E., Guo L., Schwendeman A., Li X.-A. (2015). HDL in sepsis - risk factor and therapeutic approach. *Frontiers in Pharmacology*.

[22] Guo L., Ai J. T., Zheng Z. (2013). High density lipoprotein protects against polymicrobe-induced sepsis in mice. *The Journal of Biological Chemistry*.

[23] Correa T. D., Pereira A. J., Brandt S. (2017). Time course of blood lactate levels, inflammation, and mitochondrial function in experimental sepsis. *Critical Care*.

[24] Abdulmahdi W., Patel D., Rabadi M. M. (2017). HMGB1 redox during sepsis. *Redox Biology*.

[25] Lee S.-J., Zhang J., Choi A. M. K., Kim H. P. (2013). Mitochondrial dysfunction induces formation of lipid droplets as a generalized response to stress. *Oxidative Medicine and Cellular Longevity*.

[26] Farrah T., Deutsch E. W., Omenn G. S. (2011). A high-confidence human plasma proteome reference set with estimated concentrations in PeptideAtlas. *Molecular & Cellular Proteomics*.

[27] Fjell C. D., Thair S., Hsu J. L., Walley K. R., Russell J. A., Boyd J. (2013). Cytokines and signaling molecules predict clinical outcomes in sepsis. *PLoS ONE*.

[28] Li Z., Zhang Y., Liu Y., Liu Y., Li Y. (2017). Identification of key genes in Gram-positive and Gram-negative sepsis using stochastic perturbation. *Molecular Medicine Reports*.

[29] Bertin G. I., Sabbagh A., Guillonneau F. (2013). Differential protein expression profiles between Plasmodium falciparum parasites isolated from subjects presenting with pregnancy-associated malaria and uncomplicated malaria in Benin. *The Journal of Infectious Diseases*.

[30] Su L., Zhou R., Liu C. (2013). Urinary proteomics analysis for sepsis biomarkers with iTRAQ labeling and two-dimensional liquid chromatography-tandem mass spectrometry. *Journal of Trauma and Acute Care Surgery*.

[31] Schmidt A., Forne I., Imhof A. (2014). Bioinformatic analysis of proteomics data. *BMC Systems Biology*.

