# Supplementary material for: Bioinformatical Analysis of Organ-Related (Heart, Brain, Liver, and Kidney) and Serum Proteomic Data to Identify Protein Regulation Patterns and Potential Sepsis Biomarkers
Source: Biomed Res Int. 2018 Mar 21;2018:3576157. doi: 10.1155/2018/3576157 (PMC5994327; doi:10.1155/2018/3576157)
Supplement: Supplementary Materials — Suppl. Table 1: fifty-one functions filtered by absolute number (cutoff ≥ 7) from the original 159 functions derived from GeneMania network analysis of the whole dataset without the serum proteins. Column 1 shows the functions names. Columns 2 and 3 show, respectively, the number of annotated genes in the displayed network and the number of genes with that annotation in the genome. In column 5, names in bold letters represent the genes predicted by the software. Suppl. Table 2: network analysis serum functions absolute number. Thirty-three functions filtered by absolute number (cutoff ≥ 6) from the original 166 derived from GeneMania network analysis of the serum-protein dataset. Column 1 shows the functions names. Columns 2 and 3 show, respectively, the number of annotated genes in the displayed network and the number of genes with that annotation in the genome. In column 5, names in bold letters represent the genes predicted by the software. Suppl. Table 3: subcluster 2 (gpd1, eno1, aldh5a1, coro1a, atp6v1b2, ckb, alb, fasn, acy1, fbp1, fscn1, aldh7a1, cct3, gpd1, ogdh, oxct1, and ca1). Seventeen functions filtered by prevalence (cutoff ≥ 10%) from the original 51 functions derived from GeneMania network analysis of the whole dataset without the serum proteins. Column 1 shows the functions names. Columns 2 and 3 show, respectively, the number of annotated genes in the displayed network and the number of genes with that annotation in the genome. In column 5, names in bold letters represent the genes predicted by the software. Suppl. Table 4: subcluster 2 (gpd1, eno1, aldh5a1, coro1a, atp6v1b2, ckb, alb, fasn, acy1, fbp1, fscn1, aldh7a1, cct3, gpd1, ogdh, oxct1, and ca1). Nineteen functions filtered by absolute number (cutoff ≥ 5) from the original 51 functions derived from GeneMania network analysis of the whole dataset without the serum proteins. Column 1 shows the functions names. Columns 2 and 3 show, respectively, the number of annotated genes in the displayed [file 3576157.f1.pdf]

Supplementary Material

Figures:

Suppl. Figure 1: Network analysis organs without serum.

In a GeneMANIA® network analysis each circle represents a gene. The input proteins/genes are depicted as striped circles of the same size, while the monochromatic circles, whose size is proportional to the number of interactions according to the software, can be considered as "relevant" related genes found by GeneMANIA® searching in many large, publicly available biological datasets (including protein-protein, protein-DNA and genetic interactions, pathways, reactions, gene and protein expression data, protein domains and phenotypic screening profiles). Lines linking different circles can be distinguished from their colour, mainly: violent represents co-expression (when expression levels are similar across conditions in a gene expression study); light orange represents predicted functional relationships between genes; light blue represents co-localization (when genes are expressed in the same tissue, or proteins found in the same location); light yellow represents shared protein domains (when two gene products have the same protein domain).

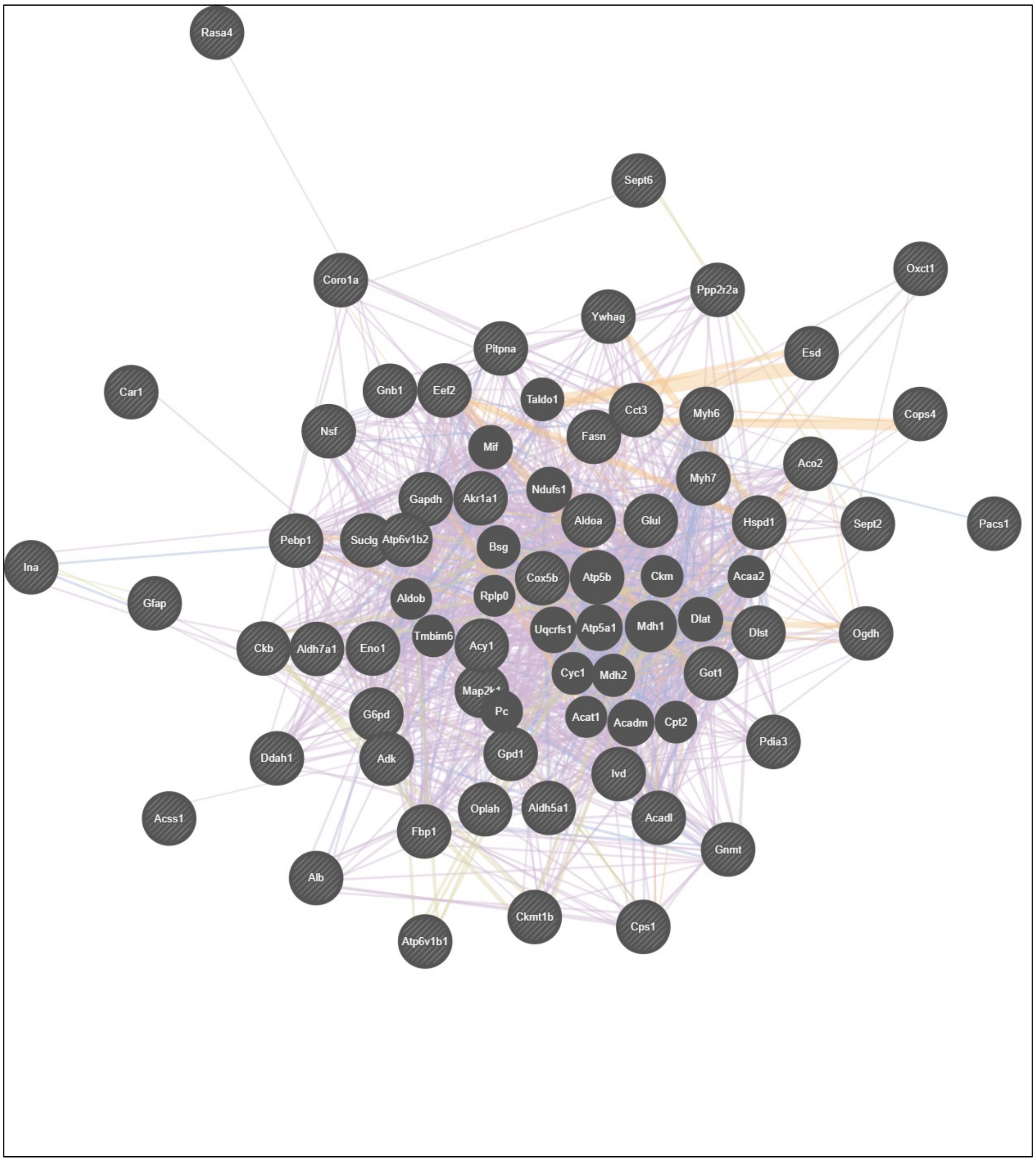
Rasa4
Sept6
Coro1a
Oxct1
Ppp2r2a
Ywhag
Pitpna
Esd
Car1
Gnb1
Eef2
Taldo1
Cct3
Myh6
Cops4
Nsf
Mif
Fasn
Aco2
Myh7
Ndufs1
Gapdh
Akr1a1
Aldoa
Glul
Hspd1
Sept2
Pacs1
Pebp1
Suclg
Atp6v1b2
Bsg
Ina
Cox5b
Atp5b
Ckm
Acaa2
Gfap
Aldob
Rplp0
Dlat
Uqcrfs1
Atp5a1
Mdh1
Dlst
Ogdh
Tmbim6
Ckb
Aldh7a1
Eno1
Acy1
Cyc1
Mdh2
Got1
Map2k
Pc
Acat1
Acadm
Cpt2
G6pd
Pdia3
Ddah1
Adk
Gpd1
Ivd
Acss1
Oplah
Aldh5a1
Acadl
Fbp1
Gnmt
Alb
Ckmt1b
Cps1
Atp6v1b1

Tables:

Suppl. Table 1: Fifty-one functions filtered by absolute number (cut-off ≥7) from the original 159 deriving from GeneMania® network analysis of the whole dataset without the serum proteins. Column 1 shows the functions names. Columns 2 and 3 show respectively the number of annotated genes in the displayed network and the number of genes with that annotation in the genome. In column 5: names in bold letters represent the genes predicted by the software.

**Function**	**Genes in network**	**Genes in genome**	**Ratio**	**Names**
generation of precursor metabolites and energy	17	196	8'67%	cps1, gnmt, ogdh, dlst, aco2, aldoa, gapdh, suclg2, eno1, fbp1, gpd1, aldh5a1, **acadm, mdh, ndufs1, mif, aldob**
coenzyme metabolic process	14	161	8'70%	Gnmt, ogdh, dlst, fasn, suclg2, g6pd, acss1, gpd1, **mdh2, mdh1, dlat, acaa2, taldo1, adob**
cofactor metabolic process	14	196	7'14%	gnmt, aldoa, akr1a1, gapdh, eno1, g6pd, fbp1, gpd1, aldh5a1, **acadm, taldo1, mif, aldob, pc**
monosaccharide metabolic process	14	199	7'04%	Gnmt, ogdh, dlst, fasn, suclg2, g6pd, acss1, gpd1, **mdh2, mdh1, dlat, acaa2, taldo1, adob**
glucose metabolic process	13	171	7'60%	gnmt, aldoa, gapdh, eno1, g6pd, fbp1, gpd1, aldh5a1, **acadm, taldo1, mif, aldob, pc**
hexose metabolic process	13	186	6'99%	gnmt, aldoa, gapdh, eno1, g6pd, fbp1, gpd1, aldh5a1, **acadm, taldo1, mif, aldob, pc**
cellular amino acid metabolic process	12	257	4'67%	gnmt, got1, glul, ckb, g6pd, ddah1, aldh5a1, ckmt1b, cps1, ivd, uroc1, **ckm**
mitochondrial inner membrane	12	269	4'46%	cps1, hspd1, cox5b, **pc, acat1, acaa2, ndufs1, atp5b, uqcrfs1, cyc1, mdh2, atp5a1**
organelle inner membrane	12	285	4'21%	cps1, hspd1, cox5b, **pc, acat1, acaa2, ndufs1, atp5b, uqcrfs1, cyc1, mdh2, atp5a1**
single-organism carbohydrate catabolic process	11	90	12'22%	cps1, aldoa, akr1a1, gapdh, eno1, c6pd, fbp1, gpd1, **taldo1, mif, aldob**
carbohydrate catabolic process	11	95	11'58%	cps1, aldoa, akr1a1, gapdh, eno1, c6pd, fbp1, gpd1, **taldo1, mif, aldob**
energy derivation by oxidation of organic compounds	11	137	8'03%	cps1, gnmt, ogdh, aco2, suclg2, gpd1, aldh5a1, dlst, **ndufs1, mdh2, acadm**
monosaccharide catabolic process	10	63	15'87%	aldoa, akr1a1, gapdh, eno1, g6pd, fbp1, gpd1, **taldo1, mif, aldob**
dicarboxylic acid metabolic process	10	76	13'16%	gnmt, ogdh, glul, suclg2, aldh5a1, got1, dlst, **mdh1, mdh2, pc**
mitochondrial matrix	10	132	7'58%	cps1, acadl, ivd, suclg2, acss1, **acadm, acaa2, atp5b, acat1, mdh2**
cofactor binding	10	148	6'76%	acadl, fasn, pitpna, g6pd, alb, gpd1, aldh5a1, **acat1, acadm, mdh1**
small molecule catabolic process	10	173	5'78%	aldh5a1, acadl, got1, oxct1, akr1a1, ivd, **acadm, cpt2, acaa2**
protein homooligomerization	10	198	5'05%	gnmt, glul, aldoa, fbp1, aldh5a1, ivd, acadl, **mif, acat1, acadm**
carboxylic acid biosynthetic process	10	200	5'00%	acss1, ckmt1b, acadl, got1, glul, fasn, akr1a1, **acadm, ckm, mif**

organic acid biosynthetic process	10	200	5'00%	acss1, ckmt1b, acadl, got1, glul, fasn, akr1a1, **acadm, ckm, mif**
adenyl ribonucleotide binding	10	289	3'46%	cps1, myh7, myh6, pebp1, fbp1, map2k1, **pc, atp5a1, atp5b**
adenyl nucleotide binding	10	293	3'41%	cps1, myh7, myh6, pebp1, fbp1, map2k1, **pc, atp5a1, atp5b**
glucose catabolic process	9	57	15'79%	fbp1, gpd1, aldoa, gapdh, eno1, g6pd, **taldo1, mif, aldob**
hexose catabolic process	9	61	14'75%	fbp1, gpd1, aldoa, gapdh, eno1, g6pd, **taldo1, mif, aldob**
coenzyme binding	9	110	8'18%	acadl, fasn, pitpna, g6pd, gpd1, aldh5a1, **acat1, acadm, mdh1**
carbohydrate biosynthetic process	9	121	7'44%	gnmt, got1, akr1a1, gapdh, g6pd, fbp1, gpd1, **pc, acadm**
carboxylic acid catabolic process	9	132	6'82%	uroc1, acadl, got1, akr1a1, aldh5a1, ivd, **acadm, cpt2, acaa2**
organic acid catabolic process	9	132	6'82%	uroc1, acadl, got1, akr1a1, aldh5a1, ivd, **acadm, cpt2, acaa2**
carboxylic acid binding	9	175	5'14%	cps1, gnmt, got1, glul, pitpna, ddah1, alb, aldh5a1, **pc**
organic acid binding	9	176	5'11%	cps1, gnmt, got1, glul, pitpna, ddah1, alb, aldh5a1, **pc**
fatty acid metabolic process	9	235	3'83%	acss1, aldh5a1, acadl, fasn, ivd, **acadm, cpt2, acaa2, mif**
ATP binding	9	261	3'45%	cps1, myh7, myh6, pebp1, fbp1, map2k1, glul, **pc, atp5a1, atp5b**
nicotinamide nucleotide metabolic process	8	43	18'60%	gpd1, dlst, ogdh, g6pd, **mdh2, mdh1, taldo1, aldob**
pyridine nucleotide metabolic process	8	43	18'60%	gpd1, dlst, ogdh, g6pd, **mdh2, mdh1, taldo1, aldob**
pyridine-containing compound metabolic process	8	51	15'69%	gpd1, dlst, ogdh, g6pd, **mdh2, mdh1, taldo1, aldob**
monosaccharide biosynthetic process	8	51	15'69%	gnmt, akr1a1, gapdh, g6pd, fbp1, gpd1, **pc, acadm**
oxidoreduction coenzyme metabolic process	8	51	15'69%	gpd1, dlst, ogdh, g6pd, **mdh2, mdh1, taldo1, aldob**
cellular respiration	8	69	11'59%	aldh5a1, ogdh, aco2, suclg2, gpd1, dlst, **ndufs1, mdh2**
cellular modified amino acid metabolic process	8	145	5'52%	cps1, gnmt, ckb, ddah1, ckmt1b, g6pd, aldh5a1, **ckm**
alpha-amino acid metabolic process	8	154	5'19%	cps1, gnmt, got1, glul, ddah1, aldh5a1, ivd, uroc1
purine ribonucleoside monophosphate metabolic process	8	199	4'02%	adk, myh7, myh6, nsf, aldoa, **atp5a1, atp5b, ndufs1**
purine nucleoside monophosphate metabolic process	8	200	4'00%	adk, myh7, myh6, nsf, aldoa, **atp5a1, atp5b, ndufs1**
ribonucleoside monophosphate metabolic process	8	203	3'94%	adk, myh7, myh6, nsf, aldoa, **atp5a1, atp5b, ndufs1**
nucleoside monophosphate metabolic process	8	214	3'74%	adk, myh7, myh6, nsf, aldoa, **atp5a1, atp5b, ndufs1**
glycolysis	7	46	15'22%	fbp1, gpd1, aldoa, gapdh, eno1, **mif, aldob**
monocarboxylic acid catabolic process	7	76	9'21%	acadl, akr1a1, aldh5a1, ivd, **acadm, cpt2, acaa2**
ATP metabolic process	7	179	3'91%	aldoa, myh7, myh6, nsf, **atp5a1, atp5b, ndufs1**
cellular carbohydrate metabolic process	7	191	3'66%	cps1, gnmt, got1, fbp1, gpd1, **acadm, mif**

response to glucocorticoid	7	218	3'21%	cps1, got1, pebp1, map2k1, **acadm, mif, aldob**
response to corticosteroid	7	232	3'02%	cps1, got1, pebp1, map2k1, **acadm, mif, aldob**
monovalent inorganic cation transport	7	283	2'47%	atp6v1b1, cox5b, nsf, **uqcrfs1, atp5a1, atp5b, mif**

Suppl. Table 2: Network analysis serum functions absolute number. Thirty-three functions filtered by absolute number (cut-off ≥6) from the original 166 deriving from GeneMania® network analysis of the serum-protein dataset. Column 1 shows the functions names. Columns 2 and 3 show respectively the number of annotated genes in the displayed network and the number of genes with that annotation in the genome. In column 5: names in bold letters represent the genes predicted by the software.

Function	Genes in network	Genes in genome	Ratio	Names
blood microparticle	22	97	22'68%	apcs, hp, c3, tf, apoa1, cfb, apoe, serping1, fga, alb, itih4, gc, **ahsg, c9, hpx, fgb, pon1, ambp, apoa2, f2, c4bpa, fgg**
glycerolipid metabolic process	9	211	4'27%	c3, apoa1, apoe, **apoa5, apoh, pon1, cps1, apoa2, apoc3**
phospholipid binding	9	222	4'05%	apoe, apoa1, **apoa5, apoh, fabp1, pon1, cps1, apoa2, apoc3**
negative regulation of hydrolase activity	9	264	3'41%	fetub, kng2, apoa1, serping1, **serpina1, fabp1, ambp, apoa2, apoc3**
lipid transport	8	174	4'60%	apoe, apoa1, **apoa5, apoh, fabp1, pon1, apoa2, apoc3**
regeneration	8	184	4'35%	fga, hp, apoa1, apoe, **apoa5, ahsg, apoa2, apoh**
enzyme inhibitor activity	8	197	4'06%	fetub, apoa1, serping1, **serpina1, ahsg, ambp, apoa2, apoc3**
wound healing	8	287	2'79%	fga, c3, apoe, **apoa5, c9, fgb, f2, apoh**
high-density lipoprotein particle	7	15	46'67%	apoe, apoa1, **apoa5, apoh, pon1, apoa2, apoc3**
plasma lipoprotein particle	7	19	36'84%	apoe, apoa1, **apoa5, apoh, pon1, apoa2, apoc3**
protein-lipid complex	7	20	35'00%	apoe, apoa1, **apoa5, apoh, pon1, apoa2, apoc3**
acylglycerol metabolic process	7	75	9'33%	c3, apoe, **apoa5, apoh, apoc3, apoa2, cps1**
neutral lipid metabolic process	7	77	9'09%	c3, apoe, **apoa5, apoh, apoc3, apoa2, cps1**
acute inflammatory response	7	96	7'29%	hp, c3, tf, itih4, serping1, **ahsg, apoa2**
lipid localization	7	136	5'15%	apoe, apoa1, **apoa5, apoh, fabp1, apoa2, apoc3**
regulation of lipid metabolic process	7	229	3'06%	c3, apoa1, apoe, **apoa5, fabp1, apoa2, apoc3**
regulation of body fluid levels	7	246	2'85%	c3, apoe, gc, fga, **c9, f2, apoh**
extracellular matrix	7	262	2'67%	apcs, alb, tf, rbp3, **f2, apoh, ahsg**
triglyceride-rich lipoprotein particle	6	14	42'86%	apoe, apoa1, **apoa5, apoh, apoc3, apoa2**
very-low-density lipoprotein particle	6	14	42'86%	apoe, apoa1, **apoa5, apoh, apoc3, apoa2**
triglyceride metabolic process	6	67	8'96%	c3, apoe, **apoa5, apoh, apoc3, cps1**
organ regeneration	6	92	6'52%	hp, apoa1, **apoa5, ahsg, apoa2, apoh**
blood coagulation	6	110	5'45%	c3, apoe, fga, **c9, f2, apoh**
hemostasis	6	112	5'36%	c3, apoe, fga, **c9, f2, apoh**
coagulation	6	115	5'22%	c3, apoe, fga, **c9, f2, apoh**
negative regulation of endopeptidase activity	6	156	3'85%	fetub, kng2, serping1, **serpina1, fabp1, ambp**

**Function**	**Genes in network**	**Genes in genome**	**Ratio**	**Names**
lipid catabolic process	6	157	3'82%	apoe, **apoa5, fabp1, cps1, apoa2, apoc3**
negative regulation of peptidase activity	6	159	3'77%	fetub, kng2, serping1, **serpina1, fabp1, ambp**
steroid metabolic process	6	200	3'00%	gc, apoa1, apoe, **apoc3, pon1, apoa2**
alcohol metabolic process	6	211	2'84%	gc, apoa1, apoe, **apoc3, pon1, apoa2**
regulation of endopeptidase activity	6	276	2'17%	fetub, kng2, serping1, **serpina1, fabp1, ambp**
organic anion transport	6	279	2'15%	dpysl2, apoa1, apoe, **apoc3, fabp1, apoa2**
regulation of peptidase activity	6	288	2'08%	fetub, kng2, serping1, **serpina1, fabp1, ambp**

Suppl. Table 3: Subcluster 2 (gpd1, eno1, aldh5a1, coro1a, atp6v1b2, ckb, alb, fasn, acy1, fbp1, fscn1, aldh7a1, cct3, gpd1, ogdh, oxct1, ca1). Seventeen functions filtered by prevalence (cut-off ≥10%) from the original 51 deriving from GeneMania® network analysis of the whole dataset without the serum proteins. Column 1 shows the functions names. Columns 2 and 3 show respectively the number of annotated genes in the displayed network and the number of genes with that annotation in the genome. In column 5: names in bold letters represent the genes predicted by the software.

Function	Genes in network	Genes in genome	Ratio	Names
NADH metabolic process	3	12	25'00%	gpd1, ogdh, **aldob**
ribonucleoside diphosphate biosynthetic process	2	10	20'00%	**atp5a1, atp5b**
carbohydrate phosphatase activity	2	10	20'00%	fbp1, **fbp2**
ADP metabolic process	2	11	18'18%	**atp5a1, atp5b**
MHC class I protein binding	2	11	18'18%	**atp5a1, atp5b**
sterol esterification	2	12	16'67%	**apoa2, scd**
cholesterol esterification	2	12	16'67%	**apoa2, scd**
steroid esterification	2	12	16'67%	**apoa2, scd**
acetyl-CoA biosynthetic process	2	13	15'38%	**acly, pdhb**
nucleoside diphosphate biosynthetic process	2	13	15'38%	**atp5a1, atp5b**
oxidoreductase activity, acting on the aldehyde or oxo group of donors	4	31	12'90%	aldh7a1, aldh5a1, ogdh, **pdhb**
NAD metabolic process	3	24	12'50%	gpd1, ogdh, **aldob**
oxidoreductase activity, acting on the aldehyde or oxo group of donors, NAD or NADP as acceptor	3	25	12'00%	aldh7a1, aldh5a1, ogdh
acetyl-CoA metabolic process	3	26	11'54%	fasn, **acly, pdhb**
gluconeogenesis	5	44	11'36%	fbp1, gpd1, **pgk1, fbp2, rbp4**
hexose biosynthetic process	5	46	10'87%	fbp1, gpd1, **pgk1, fbp2, rbp4**
glycolysis	5	46	10'87%	fbp1, eno1, gpd1, **pgk1, aldob**

Suppl. Table 4: Subcluster 2 (gpd1, eno1, aldh5a1, coro1a, atp6v1b2, ckb, alb, fasn, acy1, fbp1, fscn1, aldh7a1, cct3, gpd1, ogdh, oxct1, ca1). Nineteen functions filtered by absolute number (cut-off ≥5) from the original 51 deriving from GeneMania® network analysis of the whole dataset without the serum proteins. Column 1 shows the functions names. Columns 2 and 3 show respectively the number of annotated genes in the displayed network and the number of genes with that annotation in the genome. In column 5: names in bold letters represent the genes predicted by the software.

Function	Genes in network	Genes in genome	Ratio	Names
glucose metabolic process	8	171	4'68%	fbp1, aldh5a1, gpd1, eno1, **pgk1, fbp2, aldob, rbp4**
hexose metabolic process	8	186	4'30%	fbp1, aldh5a1, gpd1, eno1, **pgk1, fbp2, aldob, rbp4**
generation of precursor metabolites and energy	8	196	4'08%	fbp1, aldh5a1, gpd1, eno1, ogdh, **pgk1, pdhb, aldob**
monosaccharide metabolic process	8	199	4'02%	fbp1, aldh5a1, gpd1, eno1, **pgk1, fbp2, aldob, rbp4**
coenzyme metabolic process	7	161	4'35%	gpd1, fasn, ogdh, **acly, pdhb, acot2, aldob**
cofactor metabolic process	7	196	3'57%	gpd1, fasn, ogdh, **acly, pdhb, acot2, aldob**
fatty acid metabolic process	6	235	2'55%	aldh5a1, fasn, **acly, acot2, scd, fabp1**
gluconeogenesis	5	44	11'36%	fbp1, gpd1, **pgk1, fbp2, rbp4**
hexose biosynthetic process	5	46	10'87%	fbp1, gpd1, **pgk1, fbp2, rbp4**
glycolysis	5	46	10'87%	fbp1, eno1, gpd1, **pgk1, aldob**
monosaccharide biosynthetic process	5	51	9'80%	fbp1, gpd1, **pgk1, fbp2, rbp4**
acyl-CoA metabolic process	5	53	9'43%	fasn, ogdh, **acot2, pdhb, acly**
thioester metabolic process	5	53	9'43%	fasn, ogdh, **acot2, pdhb, acly**
glucose catabolic process	5	57	8'77%	fbp1, eno1, gpd1, **pgk1, aldob**
hexose catabolic process	5	61	8'20%	fbp1, eno1, gpd1, **pgk1, aldob**
monosaccharide catabolic process	5	63	7'94%	fbp1, eno1, gpd1, **pgk1, aldob**
single-organism carbohydrate catabolic process	5	90	5'56%	fbp1, eno1, gpd1, **pgk1, aldob**
carbohydrate catabolic process	5	95	5'26%	fbp1, eno1, gpd1, **pgk1, aldob**
carbohydrate biosynthetic process	5	121	4'13%	fbp1, gpd1, **pgk1, fbp2, rbp4**

Suppl. Table 5: Subcluster 3 (gapdh, cps1, aldoa, glul, myh6, myh7, oplah, got1, acss1). Twenty functions filtered by prevalence (cut-off ≥10%) from the original 90 deriving from GeneMania® network analysis of the whole dataset without the serum proteins. Column 1 shows the functions names. Columns 2 and 3 show respectively the number of annotated genes in the displayed network and the number of genes with that annotation in the genome. In column 5: names in bold letters represent the genes predicted by the software.

**Function**	**Genes in network**	**Genes in genome**	**Ratio**	**Names**
striated muscle thin filament	4	13	30'77%	**tnni1, tnnt3, tnnc2, acta1**
myofilament	4	15	26'67%	**tnni1, tnnt3, tnnc2, acta1**
actin-dependent ATPase activity	2	13	15'38%	myh6, myh7
structural constituent of muscle	3	20	15'00%	myh6, myh7, **mylpf**
ATP biosynthetic process	3	20	15'00%	aldoa, **atp5b, pkm**
microfilament motor activity	2	14	14'29%	myh6, myh7
glutamate binding	2	14	14'29%	glul, cps1
myosin II complex	2	14	14'29%	myh6, myh7
purine ribonucleoside triphosphate biosynthetic process	3	22	13'64%	aldoa, **atp5b, pkm**
purine nucleoside triphosphate biosynthetic process	3	23	13'04%	aldoa, **atp5b, pkm**
ribonucleoside triphosphate biosynthetic process	3	24	12'50%	aldoa, **atp5b, pkm**
adult heart development	2	16	12'50%	myh6, myh7
transferase activity, transferring nitrogenous groups	2	17	11'76%	got1, gapdh
regulation of the force of heart contraction	3	26	11'54%	myh6, **atp2a2, myl3**
myosin complex	3	26	11'54%	myh6, myh7, **myl3**
glycogen catabolic process	2	18	11'11%	cps1, **pygm**
cellular polysaccharide catabolic process	2	18	11'11%	cps1, **pygm**
glucan catabolic process	2	18	11'11%	cps1, **pygm**
polysaccharide catabolic process	2	18	11'11%	cps1, **pygm**
glutamate metabolic process	2	20	10'00%	got1, glul

Suppl. Table 6: Subcluster 3 (gapdh, cps1, aldoa, glul, myh6, myh7, oplah, got1, acss1). Twenty-seven functions filtered by absolute number (cut-off ≥5) from the original 90 deriving from GeneMania® network analysis of the whole dataset without the serum proteins. Column 1 shows the functions names. Columns 2 and 3 show respectively the number of annotated genes in the displayed network and the number of genes with that annotation in the genome. In column 5: names in bold letters represent the genes predicted by the software.

**Function**	**Genes in network**	**Genes in genome**	**Ratio**	**Names**
muscle system process	13	242	5'37%	myh6, myh7, aldoa, **tnnt3, actn2, atp2a2, tnni1, myl3, mybpc2, tnnc2, mylpf, acta1, mybpc1**
muscle contraction	12	198	6'06%	myh6, myh7, aldoa, **tnnt3, actn2, atp2a2, tnni1, myl3, mybpc2, tnnc2, mylpf, mybpc1**
sarcomere	8	140	5'71%	myh6, myh7, **tnnt3, tnnc2, actn2, acta1, tnni1, myl3**
contractile fiber part	8	153	5'23%	myh6, myh7, **tnnt3, tnnc2, actn2, acta1, tnni1, myl3**
myofibril	8	167	4'79%	myh6, myh7, **tnnt3, tnnc2, actn2, acta1, tnni1, myl3**
contractile fiber	8	176	4'55%	myh6, myh7, **tnnt3, tnnc2, actn2, acta1, tnni1, myl3**
adenyl ribonucleotide binding	8	289	2'77%	myh6, myh7, glul, cps1, **pkm, pygm, atp5b, atp2a2**
adenyl nucleotide binding	8	293	2'73%	myh6, myh7, glul, cps1, **pkm, pygm, atp5b, atp2a2**
ATP metabolic process	7	179	3'91%	myh6, myh7, aldoa, **pkm, atp2a2, atp5b, tnnt3**
purine ribonucleoside monophosphate metabolic process	7	199	3'52%	myh6, myh7, aldoa, **pkm, atp2a2, atp5b, tnnt3**
purine nucleoside monophosphate metabolic process	7	200	3'50%	myh6, myh7, aldoa, **pkm, atp2a2, atp5b, tnnt3**
ribonucleoside monophosphate metabolic process	7	203	3'45%	myh6, myh7, aldoa, **pkm, atp2a2, atp5b, tnnt3**
nucleoside monophosphate metabolic process	7	214	3'27%	myh6, myh7, aldoa, **pkm, atp2a2, atp5b, tnnt3**
ATP binding	6	261	2'30%	myh6, myh7, glul, cps1, **atp5b, atp2a2**
single-organism carbohydrate catabolic process	5	90	5'56%	gapdh, aldoa, cps1, **pkm, pygm**
carbohydrate catabolic process	5	95	5'26%	gapdh, aldoa, cps1, **pkm, pygm**
striated muscle contraction	5	97	5'15%	myh6, aldoa, **tnnt3, tnnc2, myl3**
ATPase activity, coupled	5	133	3'76%	myh6, myh7, **tnnt3, atp5b, atp2a2**
ATP catabolic process	5	152	3'29%	myh6, myh7, **tnnt3, atp5b, atp2a2**
purine ribonucleoside monophosphate catabolic process	5	156	3'21%	myh6, myh7, **tnnt3, atp5b, atp2a2**

Function	Genes in network	Genes in genome	Ratio	Names
ribonucleoside monophosphate catabolic process	5	156	3'21%	myh6, myh7, **tnnt3, atp5b, atp2a2**
purine nucleoside monophosphate catabolic process	5	157	3'18%	myh6, myh7, **tnnt3, atp5b, atp2a2**
nucleoside monophosphate catabolic process	5	159	3'14%	myh6, myh7, **tnnt3, atp5b, atp2a2**
ATPase activity	5	186	2'69%	myh6, myh7, **tnnt3, atp5b, atp2a2**
generation of precursor metabolites and energy	5	196	2'55%	gapdh, cps1, aldoa, **pkm, pygm**
carboxylic acid biosynthetic process	5	200	2'50%	acss1, glul, got1, **ckm, pkm**
organic acid biosynthetic process	5	200	2'50%	acss1, glul, got1, **ckm, pkm**

Suppl. Table 7: Subcluster of similarly up-regulated proteins from the serum-protein dataset (c3, kng2, dpysl2, igh-6, apoa1, hp, alb, tf, gc, apoe, cfb). Forty-four functions filtered by prevalence (cut-off ≥15%) from the original 190 deriving from GeneMania® network analysis of this subcluster dataset. Column 1 shows the functions names. Columns 2 and 3 show respectively the number of annotated genes in the displayed network and the number of genes with that annotation in the genome. In column 5: names in bold letters represent the genes predicted by the software

**Function**	**Genes in network**	**Genes in genome**	**Ratio**	**Names**
triglyceride-rich lipoprotein particle	7	14	50'00%	apoa1, apoe, **apoa4, apoh, apoc3, apoc1, apoa5**
very-low-density lipoprotein particle	7	14	50'00%	apoa1, apoe, **apoa4, apoh, apoc3, apoc1, apoa5**
high-density lipoprotein particle	7	15	46'67%	apoa1, apoe, **apoa4, apoh, apoc3, apoc1, apoa5**
phospholipid efflux	5	11	45'45%	apoa1, apoe, **apoa4, apoc3, apoc1**
plasma lipoprotein particle	7	19	36'84%	apoa1, apoe, **apoa4, apoh, apoc3, apoc1, apoa5**
protein-lipid complex	7	20	35'00%	apoa1, apoe, **apoa4, apoh, apoc3, apoc1, apoa5**
plasma lipoprotein particle remodeling	6	18	33'33%	apoa1, apoe, **apoa4, apoc3, apoc1, apoa5**
protein-lipid complex remodeling	6	18	33'33%	apoa1, apoe, **apoa4, apoc3, apoc1, apoa5**
macromolecular complex remodeling	6	18	33'33%	apoa1, apoe, **apoa4, apoc3, apoc1, apoa5**
lipase inhibitor activity	3	10	30'00%	apoa1, **apoc1, apoc3**
sterol transporter activity	3	10	30'00%	apoa1, apoe, **apoa4**
cholesterol transporter activity	3	10	30'00%	apoa1, apoe, **apoa4**
reverse cholesterol transport	3	11	27'27%	apoa1, apoe, **apoa4**
peripheral nervous system axon regeneration	3	12	25'00%	apoa1, apoe, **apoa4**
steroid esterification	3	12	25'00%	apoa1, apoe, **apoa4**
sterol esterification	3	12	25'00%	apoa1, apoe, **apoa4**
cholesterol esterification	3	12	25'00%	apoa1, apoe, **apoa4**
negative regulation of lipase activity	3	13	23'08%	apoa1, **apoc1, apoc3**
plasma lipoprotein particle organization	6	27	22'22%	apoa1, apoe, **apoa4, apoc3, apoc1, apoa5**
protein-lipid complex subunit organization	6	29	20'69%	apoa1, apoe, **apoa4, apoc3, apoc1, apoa5**
lipoprotein particle receptor binding	4	20	20'00%	apoa1, apoe, **apoc3, apoa5**
triglyceride catabolic process	3	15	20'00%	**apoc3, apoa5, apoa4**
negative regulation of fatty acid biosynthetic process	2	10	20'00%	**apoc3, apoc1**
lipase activator activity	2	10	20'00%	**apoh, apoa5**
negative regulation of cholesterol transport	2	10	20'00%	**apoc3, apoc1**
negative regulation of sterol transport	2	10	20'00%	**apoc3, apoc1**
high-density lipoprotein particle remodeling	2	10	20'00%	apoe, **apoc3**
regulation of Cdc42 protein signal transduction	3	16	18'75%	apoa1, apoe, **apoc3**
mucosal immune response	2	11	18'18%	**ltf, apoa4**

**Function**	**Genes in network**	**Genes in genome**	**Ratio**	**Names**
protein-lipid complex assembly	3	17	17'65%	apoa1, apoe, **apoa4**
regulation of sterol transport	5	29	17'24%	apoa1, apoe, **apoa4, apoc3, apoc1**
regulation of cholesterol transport	5	29	17'24%	apoa1, apoe, **apoa4, apoc3, apoc1**
phospholipid transport	6	36	16'67%	apoa1, apoe, **apoa4, fabp1, apoc3, apoc1**
regulation of lipoprotein particle clearance	2	12	16'67%	**apoc3, apoc1**
organ or tissue specific immune response	2	12	16'67%	**ltf, apoa4**
regulation of triglyceride metabolic process	4	25	16'00%	c3, **apoa5, apoa4, apoc3**
regulation of fatty acid biosynthetic process	4	25	16'00%	**apoc3, apoc1, apoa5, apoa4**
regulation of plasma lipoprotein particle levels	6	38	15'79%	apoa1, apoe, **apoa4, apoc3, apoc1, apoa5**
intestinal absorption	3	19	15'79%	apoa1, **apoa4, fabp1**
positive regulation of lipid catabolic process	3	19	15'79%	**fabp1, apoa5, apoa4**
positive regulation of fatty acid biosynthetic process	2	13	15'38%	**apoa5, apoa4**
regulation of lipoprotein lipase activity	5	33	15'15%	**apoh, apoc3, apoc1, apoa5, apoa4**
neutral lipid catabolic process	3	20	15'00%	**apoc3, apoa5, apoa4**
acylglycerol catabolic process	3	20	15'00%	**apoc3, apoa5, apoa4**

Suppl. Table 8: Subcluster of similarly up-regulated proteins from the serum-protein dataset (c3, kng2, dpysl2, igh-6, apoa1, hp, alb, tf, gc, apoe, cfb). Fifty-nine functions filtered by absolute number (cut-off ≥5) from the original 190 deriving from GeneMania® network analysis of this subcluster dataset . Column 1 shows the functions names. Columns 2 and 3 show respectively the number of annotated genes in the displayed network and the number of genes with that annotation in the genome. In column 5: names in bold letters represent the genes predicted by the software.

Function	Genes in network	Genes in genome	Ratio	Names
blood microparticle	13	97	13'40%	tf, hp, c3, apoa1, apoe, gc, cfb, alb, **ambp, itih4, ahsg, apoa4, atm**
lipid localization	9	136	6'62%	apoa1, apoe, **apoa4, rbp4, apoh, fabp1, apoc3, apoc1, apoa5**
lipid transport	9	174	5'17%	apoa1, apoe, **apoa4, rbp4, apoh, fabp1, apoc3, apoc1, apoa5**
regeneration	8	184	4'35%	hp, apoa1, apoe, **apoa4, apoh, afp, apoa5, ahsg**
glycerolipid metabolic process	8	211	3'79%	c3, apoa1, apoe, **apoa4, apoh, apoc3, apoc1, apoa5**
regulation of lipid metabolic process	8	229	3'49%	c3, apoa1, apoe, **apoa4, fabp1, apoc3, apoc1, apoa5**
triglyceride-rich lipoprotein particle	7	14	50'00%	apoa1, apoe, **apoa4, apoh, apoc3, apoc1, apoa5**
very-low-density lipoprotein particle	7	14	50'00%	apoa1, apoe, **apoa4, apoh, apoc3, apoc1, apoa5**
high-density lipoprotein particle	7	15	46'67%	apoa1, apoe, **apoa4, apoh, apoc3, apoc1, apoa5**
plasma lipoprotein particle	7	19	36'84%	apoa1, apoe, **apoa4, apoh, apoc3, apoc1, apoa5**
protein-lipid complex	7	20	35'00%	apoa1, apoe, **apoa4, apoh, apoc3, apoc1, apoa5**
triglyceride metabolic process	7	67	10'45%	c3, apoe, **apoa4, apoh, apoc3, apoc1, apoa5**
acylglycerol metabolic process	7	75	9'33%	c3, apoe, **apoa4, apoh, apoc3, apoc1, apoa5**
neutral lipid metabolic process	7	77	9'09%	c3, apoe, **apoa4, apoh, apoc3, apoc1, apoa5**
steroid metabolic process	7	200	3'50%	gc, apoa1, apoe, **apoa4, afp, apoc3, apoc1**
phospholipid binding	7	222	3'15%	apoa1, apoe, **apoa4, apoh, fabp1, apoc3, apoa5**
negative regulation of hydrolase activity	7	264	2'65%	apoa1, kng2, **fabp1, ambp, serpina1, apoc3, apoc1**
organic anion transport	7	279	2'51%	dpysl2, apoa1, apoe, **apoa4, fabp1, apoc3, apoc1**
plasma lipoprotein particle remodeling	6	18	33'33%	apoa1, apoe, **apoa4, apoc3, apoc1, apoa5**
protein-lipid complex remodeling	6	18	33'33%	apoa1, apoe, **apoa4, apoc3, apoc1, apoa5**
macromolecular complex remodeling	6	18	33'33%	apoa1, apoe, **apoa4, apoc3, apoc1, apoa5**
plasma lipoprotein particle organization	6	27	22'22%	apoa1, apoe, **apoa4, apoc3, apoc1, apoa5**
protein-lipid complex subunit organization	6	29	20'69%	apoa1, apoe, **apoa4, apoc3, apoc1, apoa5**
phospholipid transport	6	36	16'67%	apoa1, apoe, **apoa4, fabp1, apoc3, apoc1**

**Function**	**Genes in network**	**Genes in genome**	**Ratio**	**Names**
regulation of plasma lipoprotein particle levels	6	38	15'79%	apoa1, apoe, **apoa4, apoc3, apoc1, apoa5**
organophosphate ester transport	6	53	11'32%	apoa1, apoe, **apoa4, fabp1, apoc3, apoc1**
lipid transporter activity	6	55	10'91%	apoa1, apoe, **apoa4, rbp4, fabp1, apoc3**
regulation of lipase activity	6	74	8'11%	apoa1, **apoc1, apoa5, apoa4, apoh, apoc3**
organ regeneration	6	92	6'52%	hp, apoa1, **apoa5, ahsg, apoh, afp**
regulation of lipid biosynthetic process	6	118	5'08%	c3, apoe, **apoa4, apoc3, apoc1, apoa5**
cellular ketone metabolic process	6	145	4'14%	**afp, apoc3, apoc1, apoa5, apoa4, fabp1**
lipid catabolic process	6	157	3'82%	apoe, **apoa4, fabp1, apoc3, apoc1, apoa5**
enzyme inhibitor activity	6	197	3'05%	apoa1, **apoc1, ahsg, ambp, serpina1, apoc3**
alcohol metabolic process	6	211	2'84%	gc, apoa1, apoe, **apoa4, apoc3, apoc1**
enzyme activator activity	6	225	2'67%	apoa1, apoe, **apoa4, apoh, ltf, apoa5**
phospholipid efflux	5	11	45'45%	apoa1, apoe, **apoa4, apoc3, apoc1**
regulation of sterol transport	5	29	17'24%	apoa1, apoe, **apoa4, apoc3, apoc1**
regulation of cholesterol transport	5	29	17'24%	apoa1, apoe, **apoa4, apoc3, apoc1**
regulation of lipoprotein lipase activity	5	33	15'15%	**apoh, apoc3, apoc1, apoa5, apoa4**
cholesterol efflux	5	36	13'89%	apoa1, apoe, **apoa4, apoc3, apoc1**
regulation of lipid catabolic process	5	42	11'90%	**fabp1, apoc3, apoc1, apoa5, apoa4**
cholesterol transport	5	55	9'09%	apoa1, apoe, **apoa4, apoc3, apoc1**
sterol transport	5	55	9'09%	apoa1, apoe, **apoa4, apoc3, apoc1**
cholesterol homeostasis	5	57	8'77%	apoa1, apoe, **apoa4, apoc3, apoa5**
sterol homeostasis	5	58	8'62%	apoa1, apoe, **apoa4, apoc3, apoa5**
regulation of fatty acid metabolic process	5	59	8'47%	**fabp1, apoc3, apoc1, apoa5, apoa4**
regulation of lipid transport	5	75	6'67%	apoa1, apoe, **apoa4, apoc3, apoc1**
cholesterol metabolic process	5	80	6'25%	apoa1, apoe, **apoa4, apoc3, apoc1**
sterol metabolic process	5	81	6'17%	apoa1, apoe, **apoa4, apoc3, apoc1**
lipid homeostasis	5	87	5'75%	apoa1, apoe, **apoa4, apoc3, apoa5**
acute inflammatory response	5	96	5'21%	tf, hp, c3, **itih4, ahsg**
regulation of cellular ketone metabolic process	5	102	4'90%	**fabp1, apoc3, apoc1, apoa5, apoa4**
positive regulation of lipid metabolic process	5	106	4'72%	apoa1, apoe, **apoa4, fabp1, apoa5**
cellular lipid catabolic process	5	123	4'07%	**fabp1, apoc3, apoc1, apoa5, apoa4**
organic hydroxy compound transport	5	140	3'57%	apoa1, apoe, **apoa4, apoc3, apoc1**
tissue homeostasis	5	170	2'94%	tf, alb, **ahsg, rbp4, ltf**
anatomical structure homeostasis	5	224	2'23%	tf, alb, **ahsg, rbp4, ltf**
fatty acid metabolic process	5	235	2'13%	**fabp1, apoc3, apoc1, apoa5, apoa4**
transition metal ion binding	5	239	2'09%	tf, alb, **apoa4, meltf, ltf**
